# Towards sentiment aided dialogue policy learning for multi-intent conversations using hierarchical reinforcement learning

**DOI:** 10.1371/journal.pone.0235367

**Published:** 2020-07-02

**Authors:** Tulika Saha, Sriparna Saha, Pushpak Bhattacharyya

**Affiliations:** Department of Computer Science and Engineering, Indian Institute of Technology Patna, India; Lingnan University, HONG KONG

## Abstract

**Purpose:**

Developing a Dialogue/Virtual Agent (VA) that can handle complex tasks (need) of the user pertaining to multiple intents of a domain is challenging as it requires the agent to simultaneously deal with multiple subtasks. However, majority of these end-to-end dialogue systems incorporate only user semantics as inputs in the learning process and ignore other useful user behavior and information. Sentiment of the user at the time of conversation plays an important role in securing maximum user gratification. So, incorporating sentiment of the user during the policy learning becomes even more crucial, more so when serving composite tasks of the user.

**Methodology:**

As a first step towards enabling the development of sentiment aided VA for multi-intent conversations, this paper proposes a new dataset, annotated with its corresponding intents, slot and sentiment (considering the entire dialogue history) labels, named *SentiVA*, collected from open-sourced dialogue datasets. In order to integrate these multiple aspects, a Hierarchical Reinforcement Learning (HRL) specifically *options* based VA is proposed to learn strategies for managing multi-intent conversations. Along with task success based immediate rewards, sentiment based immediate rewards are also incorporated in the hierarchical value functions to make the VA user adaptive.

**Findings:**

Empirically, the paper shows that task based and sentiment based immediate rewards cumulatively are required to ensure successful task completion and attain maximum user satisfaction in a multi-intent scenario instead of any of these rewards alone.

**Practical implications:**

The eventual evaluators and consumers of dialogue systems are users. Thus, to ensure a fulfilling conversational experience involving maximum user satisfaction requires VA to consider user sentiment at every time-step in its decision making policy.

**Originality:**

This work is the first attempt in incorporating sentiment based rewards in the HRL framework.

## 1 Introduction

### Contextualization

Goal-oriented Dialogue System continues to be an area of immense interest for the NLP researchers and AI in particular where VAs in the form of rational agents have to complete a predefined goal or retrieve information (related to booking of flights, restaurants etc.) by interacting with users via natural language. Prominent works in the context of Dialogue Management (DM) include those of [1–9] etc. But such works lack diversity, i.e., those works are related to the context of serving a particular dialogue scenario or intent of the user. But in the real world applications, user generally wants to accomplish tasks which include getting several intents/subtasks fulfilled in a single dialogue conversation with minimal effort and dialogue turns. Thus, creating VAs to manage composite goal of the user pertaining to multi-intent conversations in an unified manner is the need of the hour.

### Relevance

Also, the eventual evaluators and consumers of such dialogue systems are users. So, for a fulfilling conversational experience involving maximum user satisfaction requires VA to consider user sentiment at every time-step in its decision making policy. The extra feedback from the user in terms of sentiment will steer the VA to be user adaptive in order to learn an efficient dialogue policy [10] as user sentiment is a true reflection of user satisfaction. VAs of such kind become immensely important in today’s time where the demand for such automated and personalized VAs is at an ever high. What makes these creation of VAs even more difficult is the complexity involved as the VA needs to solve composite queries of user in a single dialogue conversation while also taking care of the user’s sentiment.

### Research question

Reinforcement Learning (RL) [11, 12] has been utilized over the years to solve the problem of dialogue management and it has been proven to be quite effective to model the above task by treating it as an optimization problem. But as the ever-growing needs and the complexities of the user are taken into consideration, there arises an imperative need to curate comprehensive dialogue managers. These dialogue managers should be capable of handling larger and intricate state space of dialogues, supervise multiple dialogue scenarios with ease, higher accuracy and precision. These intense challenges make traditional RL models [1, 13–16] unscalable to manage such complex conversations. Hierarchical RL (HRL) [17–19] on the contrary provides a more doctrined way for learning dialogue management strategies or policies for complex problems. It focuses on reducing the problem of curse of dimensionality that afflicts the creation and modeling of solutions for such complex tasks by dividing a composite task into numerous and sequence of subtasks. Thus, it needs to be studied and evaluated that how HRL can be employed to provide a learning framework that caters to the requirement of handling various subtasks at the same time while additionally also taking into account other behavioral cues of the user such as sentiment to serve the user in an efficient manner.

### Objective

This paper proposes a HRL framework specifically *options* based VA to model the task of learning dialogue policies for multi-intent conversations for successful task completion. Along with it, user’s sentiment is also incorporated into the hierarchical value functions to attain maximum user satisfaction. A unique representation of Semi-MDP is proposed with novel task based and sentiment based reward functions to guide the learning process of the VA. To address all these aspects together, a new dataset is introduced which is collected from open-source dialogue datasets containing multi-intent conversations with sentiment pertaining to *Restaurant* domain. Empirically, it is shown that apart from user semantics, additional user behavioral information such as sentiment plays an important role in attaining maximum user satisfaction while creating complex VAs of composite nature.

The key contributions of this paper are the following:

- Integration of hierarchical value functions with Deep Reinforcement Learning (DRL) for the VA to learn strategies for managing multi-intent conversations. Along with it, sentiment of the user is incorporated to these hierarchical value functions to make the VA adaptive to the sentiment of the user.
- It is shown empirically that task based and sentiment based immediate rewards cumulatively are required to ensure successful task completion and attain maximum user satisfaction in a multi-intent scenario instead of these rewards alone.
- First large scale dataset named **SentiVA** for multi-intent conversations annotated with its corresponding intent, slot and sentiment (considering the entire dialogue history) labels for the Restaurant domain is made available.

### Structure of the paper

Section 2 presents a brief overview of the recent works for RL based Dialogue Management Strategy followed by the motivation behind solving the current problem. Section 3 demonstrates the process of dataset creation and its details. Section 4 presents the proposed methodology in detail. Section 5 lists the experimental details for the implementation of the proposed methodology. Section 6 presents all the experimental results along with its detailed discussion and error analysis. Section 7 presents the conclusion and the future works.

## 2 Related work

### 2.1 Background

In recent times, two prominent paradigms of research have emerged in Goal-oriented Dialogue Systems. The first category includes sequence to sequence based supervised models [20], encompassed as Natural Language Generation (NLG) task wherein an user utterance and its context are encoded to decode a VA response directly [21]. The data requirement for these categories of models is huge as they directly imitate the knowledge contained within the training data [8]. The second ones are frameworks based on Reinforcement Learning (RL) algorithms such as Deep Q-Networks (DQN) [22] wherein supervised learning techniques are combined and applied to RL tasks [14]. These approaches require less amount of data as compared to the former because of their ability to simulate dialogue conversations. They explore various facets of dialogue space efficiently by exploiting its sequential nature. The focus of this paper is on the latter category for developing VAs for what is popularly known as the Dialogue Management (DM) task.

The concept of HRL is relatively old with some of its works that date back to the early 1970s. [23] proposed a HRL approach based on options framework to learn policies in different domains. In [24], authors propose a divide and conquer approach for efficient policy learning where a complex goal-oriented task is broken into simpler subgoals in an unsupervised manner and then these subgoals are used to learn a multi-level policy using HRL. Feudal Reinforcement Learning has been used with DQN in the work of [25] for learning policies in large domains. These works are significantly different in their problem statements where the focus was to propose DM methodologies to handle multi-domain conversations with a single subtask/intent per domain. Whereas our work focuses on handling multi-intent dialogue conversations pertaining to a single domain. In [10], authors used only sentiment based immediate rewards in an end-to-end dialogue system for a single intent.

Apart from them, there are other significant works that aim to propose methodologies to learn DM policies for a single intent pertaining to a domain. In [1], authors developed an easy and open-sourced dialogue system using DRL for the restaurant domain and so the system evades from using hand-crafted features for learning an action-selection strategy without the use of the Natural Language Understanding (NLU) module. It employs the Deep Q-Network (DQN) algorithm for its implementation. One of the limitations of this work is that even if the VA learns an optimal policy, its usability is restricted because of its dependence on the vocabulary. The system falters in out of vocabulary words and hence is difficult to be scalable in complex scenarios. In [26], authors proposed a fast DRL approach that uses a network of DQN agents that skips weight updates during exploitation of actions. In [6], authors proposed a variant of DQN where the VA explores via Thompson sampling, drawing Monte Carlo samples from a Bayes-by-Backprop neural networks by maintaining a probability distribution over the weights in the network. In [7], authors presented an adversarial advantage actor-critic based model that comprises of a discriminator to differentiate actions generated by VAs from actions by experts. Later, a discriminator is also added as another critic into the framework to encourage VAs to explore state-action within the regions where the agent takes action identical to those of the experts.

In [27], authors presented a Hindsight Experience Replay (HER) based Dialogue Policy Learning from failed conversations. They tuned the vanilla ER to incorporate two other types of ER mechanisms namely Trimming based HER that trims failed conversations to generate successful ones and Stitching based HER that computes the similarity between belief states and stitch together segments to create successful dialogues. They formulate their problem statement for a movie booking task as a Markov Decision Problem (MDP) and demonstrate their proposed approach using DQNs. In [28], authors developed a DRL based approach on Dyna-Q framework, where they introduced a world model to simulate the environment. So, now the VA learns from direct RL method using real experiences from the data and also from the simulated user experience generated from the world model (which is multi-classification network: two classification and one regression task to simulate several aspects such as user action, rewards etc.) to combat the absence of large conversational data for training. They also formulated their problem statement for a movie booking task as a Markov Decision Problem (MDP) and demonstrated their proposed approach using DQNs. In [29], authors extended the work of Deep Dyna-Q framework [28] to counter the low quality of simulated user experience from the world model. They incorporated a discriminator (influenced from adversarial network) to differentiate between the real user experience and the simulated ones. The ones where the discriminator failed to identify or had difficulty detecting the simulated experiences from the real ones were then used in the policy learning phase of the VA. In [30], authors presented yet another variant of Deep Dyna-Q framework [28] called Switch-based Active Deep Dyna-Q to counter the problem of low quality simulated user experience of the world model and the sample efficiency of the Dyna-Q framework. They incorporated a switcher and an active sampling strategy to determine when to use real or simulated user experience depending on different phases of dialogue policy training and generate those simulated user experiences that have not been fully explored by the VA. In [31], authors presented yet another variant of Deep Dyna-Q framework [28] called Budget-Conscious Scheduling-based (BCS) Deep Dyna-Q to best utilize a fixed, small number of human interactions (budget) for learning dialogue policies. They incorporated a BCS module to manage the budget and select the most effective way to generate real or simulated experiences for learning a dialogue policy in a fixed budget. In all these works stated above, the focus was on proposing different ways combined with DQN that requires lesser real user experience for training VA for a single intent of movie booking task. Additionally in [31], author’s aim was to demonstrate that how in fixed budget setting (limited human experience) a cost-effective dialogue policy can be learnt as obtaining high quality dialogue data is a challenging task in itself. Thus, they proposed different ways to tweak the DQN algorithm to incorporate different aspects related to their task at hand. However, the current work focuses on incorporating sentiment, an important user behavior in the learning process to handle multi-intent conversations with the help of HRL.

Independently, there exists several works in literature focused on developing supervised and unsupervised models for understanding sentiment from user utterance [32–34]. However, there exists very little work that utilizes these additional information of the user behavior in the decision making process for the VA to be efficient and competent enough to converse and execute its goal appropriately. In [35–37], authors used rule-based reactions to incorporate sentiment as a part of dialogue policies in order to create interpersonal interactions. In [10], authors used sentiment based rewards instead of task success based rewards in the policy learning process to establish that sentiment provides better reward assistance for the VA to achieve user goal. However, their work is focused on learning dialogue policy for only a single intent throughout the conversation. But in a real-life scenario such as multi-intent and multi-domain, sentiment based rewards alone can’t solve the purpose as apart from keeping user sentiment in mind there also exists other complexities such as sub-task, multi-task completion etc.

### 2.2 Motivation

From the literature, it is evident that several works done earlier in the context of dialogues had shortcomings. The applications developed earlier based on the traditional RL approach had tremendous amount of human labor and interference involved right from manual hand-crafting of the rules to carrying out experiments to train the agent. Performing large scale experiments to establish the robustness of the learnt strategy was a cumbersome process. State tracking was difficult because the representation of the states in the MDP was complex as more number of variables with varied ranges were used to capture the information in a particular time-step. Recent research focuses on merging the NLU and DM into a single module eliminating the need of NLU modules and creating a single model in order to avoid NLU fault chances. These types of models restrict the usability of trained policy only to situations where dialogue vocabulary matches the training corpus, change in vocabulary requires a new model to be trained from scratch which becomes cumbersome for continually evolving and online systems. Recent works, which employed Deep RL techniques for the problem, incorporate vocabulary of the system as state representation without the use of NLU module. So, even if the VA learns an optimal policy, its usability is restricted because of its dependence on the vocabulary and hence is not scalable. Other approaches proposed require extensive dialogue data, demand huge computational cost for training such complex networks. Often scalability, reusability and reproducibility of these proposed models are not achievable in real life implementation scenarios. Apart from user semantics, other useful information such as sentiment that depicts an aspect of user behavior were never integrated in the learning process to address multi-intent scenarios. Also, majority of these works focus on serving single intent or task of the user in a dialogue conversation which is highly not desirable in practical scenarios.

Motivated by the inadequacy of the existing systems and approaches, this paper presents an approach to serve multiple intents of the user in a single dialogue conversation without discretizing information across intents using Hierarchical Deep Reinforcement Learning. Apart from these, sentiment based rewards is also incorporated along with task success based rewards for the VA to understand and mimic human behavior as closely as possible and provide gratifying user experience and satisfaction.

## 3 Dataset

To facilitate the research in dialogue policy learning assisted with user sentiment pertaining to multiple intents, this paper introduces a new dataset (SentiVA) consisting of dialogue conversations manually annotated with its intent, slot and sentiment (considering the entire dialogue history) labels.

### Data collection

For the current work, the dialog bAbI dataset [2] is used to curate conversations for the *SentiVA* dataset. The dialog bAbI dataset contains conversations for a set of 6 tasks for testing end-to-end dialog systems in the *Restaurant* domain. Each task tests a unique aspect of dialog. For each task, there are 1000 dialogue for training, 1000 for development and 1000 for testing. This particular dataset was chosen because of its task-oriented nature and also conversations were primarily based on slot-filling structure with user satisfaction taken into account. This dataset contains conversations concerning several intents such as *restaurant_info*, *restaurant_book*, *restaurant_phone*, *restaurant_address* involving slots or entities such as <*location*>, <*cuisine*>, <*no. of people*>, <*restaurant name*> and <*price*>. Dialogues pertaining to task-4 and 5 were utilized to prepare conversations for the current work. To the best of our knowledge, we were unaware of any sizable and open-access dialogue data pertaining to multi-intent conversations annotated with its corresponding intent, slot and especially sentiment labels at the time of writing. Thus, dialog bAbI dataset has been manually modified and annotated for the corresponding intent, slot and sentiment labels to make it suitable for developing a VA capable of learning strategies to converse with the user to accomplish its composite task by taking into account user sentiment and enable novel research in the field of sentiment aided dialogue policy learning.

### Data annotation

Initially, each conversation was modified to incorporate multiple intents such as the combination of intents mentioned above to a maximum of three intents per dialogue. Then each of these conversations were annotated for their corresponding utterance-level intents and word-level slots. Following this, conversations were also annotated for the sentiment into three categories, i.e., positive, negative and neutral. For the annotation of sentiment, the annotators were presented with the entire dialogue history and were explicitly asked to focus on the user’s conduct and nature rather than that of the VA’s. Three annotators graduate in English linguistics were assigned the task of annotating the sentiments. The inter-annotator score (kappa) with more than 0.80 was considered as reliable agreement.

### SentiVA dataset

**Fig 1:**
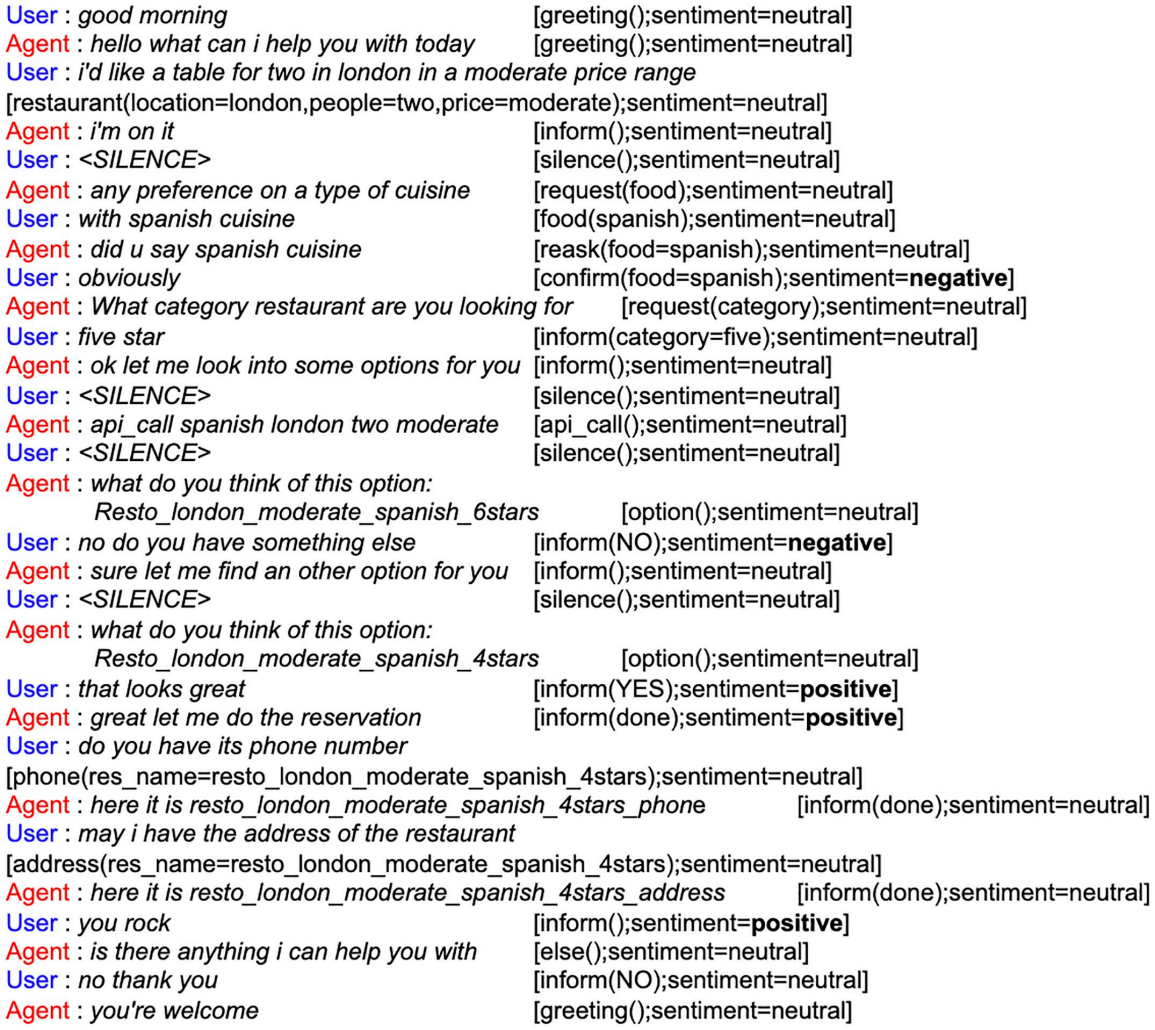
A sample chat transcript from the annotated dataset.

The *SentiVA* dataset contains a total of 1286 dialogues modified for the presence of multi-intents in a particular dialogue and annotated for its corresponding intent, slot and sentiment labels. Tables 1 and 2 show statistics of the annotated dataset. The skewness in the dataset for sentiment (as seen in Table 2) can be attributed to the nature of the task. In task-oriented scenarios, users are less likely to depict negative or positive sentiments unless an extraordinary circumstance. Fig 1 shows a sample chat transcript from the annotated dataset with its corresponding intent, slot and sentiment labels. To the best of our knowledge, this dataset, *SentiVA* is the first large scale, open-access dataset for multi-intent conversations annotated with its corresponding intent, slot and sentiment (considering the entire dialogue history) labels. In [10], authors annotated conversations considering the dialogue history for conversations pertaining to single goal or intent. In [38], authors also created a similar dataset with emotion labels for a single intent but those are not annotated considering the conversation context.

**Table 1:** Statistics of the developed dataset.

Category	# Dialogues	# Utterances
Train	750	23500
Development	182	2967
Test	350	8800

**Table 2:** Sentiment label distribution across the annotated dataset.

Category	# Utterances
Neutral	31231
Positive	2860
Negative	1176

### Qualitative analysis

The current work seeks to analyze the effect of sentiment in learning dialogue strategies for the VA while also taking into account the goal of accomplishing composite task of the user pertaining to multiple intents of a domain in a single dialogue conversation. Below, an analysis is provided using instances from the proposed dataset to further support the claim which requires sentiment aided reasoning along with multi-intent conversation illustration.

- **Role of multi-intent conversation**: In real-life scenario, users generally take assistance of VA in order to fulfill its complex and composite goal and do not restrict themselves to just one task per conversation. Thus, incorporating such complex scenarios in dialogue conversations is the need of the hour in order to make the VA more competent and efficient in handling such events. Here, multi-intent means addressing more than one intention of the user across the dialogue. As seen in Fig 1, the VA handles such scenarios by addressing multiple intents across the dialogue as *restaurant*, *phone* and *address*.
- **Role of user sentiment**: As explained above, incorporating user sentiment in the learning process helps attain maximum user gratification. As seen in Fig 1, the *negative* sentiment of the user helped the VA in providing more options to the user for its satisfaction. Otherwise its task of successfully filling up all relevant slots along with a valid database query is attained in the first *option* turn itself. However, the user wasn’t satisfied with the provided option and that is only visible from its sentiment. Also, different cases where sentiment actually plays a role include *repetition*, *interruption* [10] in every sub-task and completion of each of the subtask successfully as queried by the user to achieve the goal in whole to attain maximum or absolute user satisfaction. *Repetition* are primarily of two types: *(i)*. where the user asks the VA to reiterate its prior action or utterance; *(ii)*. where the VA falls in a loop asking or picking up the same action continuously due to its failure to understand some entity corresponding to the intent being served. *Interruption* means the user interrupting the VA while it is processing a subgoal or a subtask. Note that subtask and subgoal has been used synonymously in this paper.

## 4 Materials and methods

This paper employs a well known foundation of HRL called the *Options*, belonging to the group of decision problems called the Semi-MDPs [17]. Options framework fundamentally provides a hierarchical schema to decompose a composite task into several subtasks at different levels of hierarchies. Thus, we integrate hierarchical value functions with DRL for the VA to learn strategies for managing multi-intent conversations in an unified manner. Along with it, sentiment of the user is incorporated to these hierarchical value functions to ensure higher user satisfaction and make the VA adaptive to the sentiment of the user.

### Hierarchical DRL Agent

It is a two-level HDRL agent that comprises of a top-level intent meta-policy, *π*_*i*,*d*_ and a low-level controller policy, *π*_*a*,*i*,*d*_. The intent meta-policy takes as input state *s* from the environment and selects a subtask *i* ∈ *I* among-st multiple subtasks identified based on the user requirement, where *I* represents the set of all intents/subtasks of that domain. The controller policy and its state space *π*_*a*,*i*,*d*_ are shared amongst all the options/intents thereby satisfying slot constraints amongst overlapping subtasks. It inputs state *s* and outputs a sequence of primitive actions *a* ∈ *A* where *A* represents the set of all the primitive actions of a domain. The internal critic present in the VA gives task based immediate rewards to top and low-level policies, respectively, at every time-step for picking up actions at different points in the conversation to ensure successful task completion. To conceive the HDRL agent, a generic architecture of semi-MDP is used. It finds its applicability in any domain having *n* number of intents and *m* number of slots.

### Incorporating sentiment

**Fig 2:**
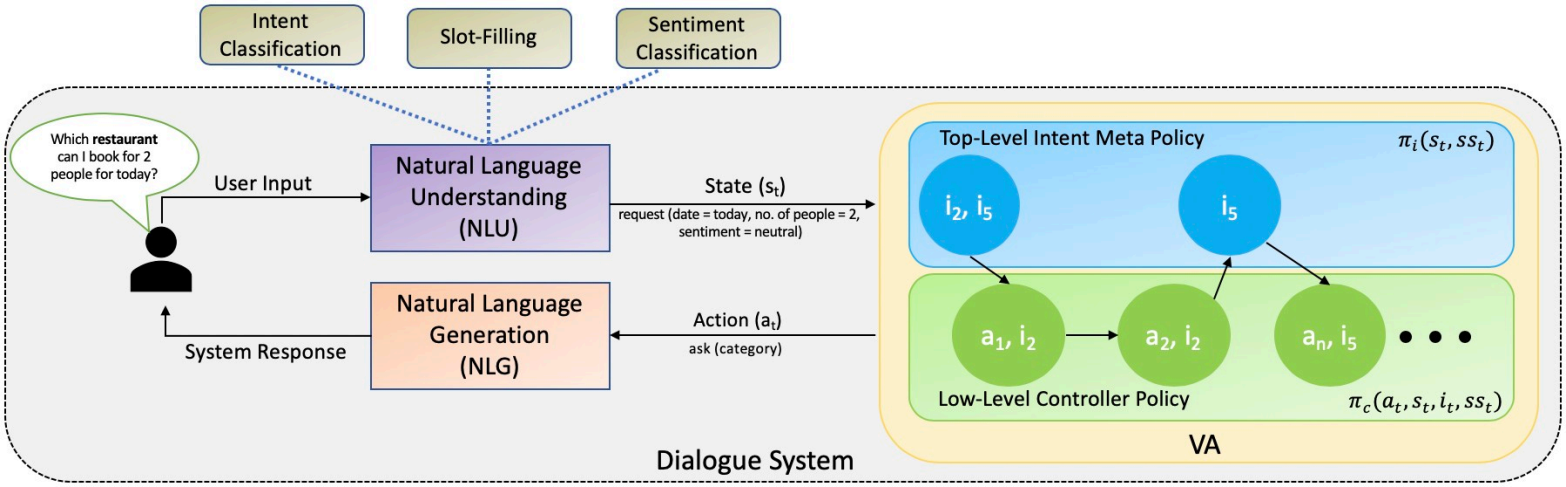
End-to-end framework for a two-level proposed hierarchical dialogue manager fused with sentiment (*ss*).

Primarily, works in literature are focused on improving techniques or dialogue policies for the VA to be diverse enough to handle complex scenarios (such as multi-intent conversations) for task (user goal) fulfillment such as [23, 25]. As a result only task success based immediate rewards were incorporated in the training phase for Reinforcement Learning (RL) based algorithms for the VA to learn policies. In this work, the focus is in integrating sentiment based immediate rewards (identified from the user utterance) with immediate rewards from the internal critic to assure higher user satisfaction and better user experience. To accomplish this, a novel reward function is proposed that fuses user sentiment so that it emulates or mimics human behavior. So as mentioned above, these sentiment based rewards will be incorporated in the hierarchical value functions to ensure higher user satisfaction and make the VA adaptive to the sentiment of the user. What the VA really needs to distinguish is the negative cases from the neutral and positive cases as the dialogue evolves, in order to avert negative sentiment and end the conversation on a positive note for the user. Therefore, the user sentiment scores at every time-step of the conversation is detected using the sentiment classifier/detector already pre-trained using the dataset discussed above on the fly and used them in the state space and the reward models of the semi-MDP for an end-to-end dialogue training. Different cases where sentiment actually plays a role include *repetition*, *interruption* [10], *user satisfaction* in every sub-task to cumulatively complete entire task (multi-intents) designated by the user. The utility of integrating these notions will be explained empirically in later sections. Fig 2 shows the architectural diagram of the proposed Hierarchical DRL agent fused with sentiment.

### State space

An universal state space for both the intent meta and controller policies is used which is a tuple of *n* + *m* + 1 variables to a total of *z* variables. For the intent meta-policy, the *n* variables are multi-hot encoding values representing the multi-label intents identified by the pre-trained Intent Classification (IC) module for a given user utterance. Whereas for the controller policy, the *n* variables are one-hot encoding values representing the current option/intent being picked up by the intent meta-policy to be served. *m* variables store the confidence scores of different slots which are the probability values outputted from the pre-trained Slot-Filling (SF) module, representing the confidence of the module in predicting different slot labels. The task of the controller policy is to then pick up primitive actions to fill in relevant slots from *m* pertaining to the option in control. The *z*th variable corresponds to the user’s sentiment score (*ss*) which is the probability value (*P*_*s*_) outputted from the pre-trained Sentiment Classification (SC) module for a given user utterance.

$$\begin{array}{c} S_{z}=\{\begin{array}{cc} P_{s}, & \;\text{where}\;P_{s}>0.5\;\text{if}\;tag=+ve\\ 1-P_{s}, & \;\text{where}\;1-P_{s}<0.5\;\text{if}\;tag=-ve\\ 0.5, & \;\text{otherwise}\; \end{array} \end{array}$$

### Action space

The action space consists of actions for the meta as well as controller policies. For the intent meta-policy, *n* + 1 options are available to serve the intents. The *n* + 1th option represents an option to execute the policy to ask the user if he/she needs any other services from the VA when all the tasks previously queried by the user are successfully completed. For the controller policy, 21 primitive actions are available; categorized in five different classes, i.e., *Ask*, *Reask/Confirm*, *Update*, *Option* and *Salutation*.

### Reward model

The task based and sentiment based reward functions for different hierarchies at different time-steps of the dialogue are as follows:

- **Controller policy**: The task based reward (*TR*_*c*_), at every time-step of the conversation (*TR*_*c*_(*s*, *a*, *i*, *s*′)) for the controller policies is as follows:
  $$\begin{array}{c} TR_{c}=\{\begin{array}{c} \left(w_{1}*\left(\|\vec{S^{\prime }}{\|}_{1}\;-\;\|\vec{S}{\|}_{1}\right)\right)-\left(w_{2}\right),\;\text{if}\;a=nta\\ w_{1}*\|\vec{S}{\|}_{1},\;\;\;\text{if}\;a=ta\;\&check\left(S\right)=True\\ -w_{1}*\left(\|\vec{EV}{\|}_{1}-\|\vec{S}{\|}_{1}\right),\;\text{if}\;a=ta\\ \;\;\;\;\;\;\;\;\;\;\;\;\;\&check(S)=False \end{array} \end{array}$$
  where *nta* = non-terminating action, *ta* = terminating action. $\|\vec{S^{\prime }}{\|}_{1}$ is the summation of the confidence scores of all the state variables in the state vector $s^{\prime^{\prime }}$ which is obtained after taking an action *a* in state *s*. $\|\vec{S}{\|}_{1}$ is the summation of the confidence scores of all the state variables in the state vector *s*. *w*_1_ encourages the agent to act in a way so as to increase its confidence on the acquired slots. *w*_2_ encourages useful communication and discourages unnecessary iterations. Here, *w*_1_ = no. of unique slots of the domain and *w*_2_ = 1 for our experiments. All specific values of *w*_1_ and *w*_2_ were assigned through empirical analyses by conducting the parameter sensitivity tests. $\|\vec{EV}{\|}_{1}$ is the summation of the maximum expected confidence scores of different slots that adds up to be equal to *m* for controller policies with *m* slots (the maximum expected confidence score for each slot being 1). The checking criteria (*check*(*s*)) is as follows: if the confidence scores of all the individual slots for a particular controller state *S* ≥ *threshold* (set to 0.7) are relevant to the option in control, then the checking condition is *True*, otherwise it is *False*.

### Case study

Let $\vec{S}$ be—1 0 0 0 0.0 0.0 0.0 0.83 0.0 0.5—(say) at a particular time-step and $\vec{S^{\prime }}$ be—1 0 0 0 0.0 0.0 0.91 0.83 0.0 0.5—(say) at the next time-step by picking up a correct *nta* i.e., a non-terminating action (say ask(no. of people)). So *TR*_*c*_ = 5 * (1.74 − 0.83) − 1, i.e., a positive reward of 3.55. Also, for e.g., let’s say an incorrect *nta* was picked up not relevant to the current option (say ask(price)). In that case, *nta* doesn’t have any effect in the state space, so, $\vec{S^{\prime }}$ remains |1 0 0 0 0 0 0 0.83 0 0.5|. Then, *TR*_*c*_ = 5 * (0.83 − 0.83) − 1, thereby, imposing a penalty of −1. Now, for eg., in state $\vec{S}$—1 0 0 0 0.0 0.0 0.91 0.83 0.0 0.83—(say), the VA picks up the *ta*. Here, *check*(*S*) becomes *True*, as all the slots pertaining to the option in control are elicited with higher confidence. So, *TR*_*c*_ = 5 * 1.74, i.e., a high positive reward of 8.7. Now, let’s say that the VA picks up a *ta* in state $\vec{S}$—1 0 0 0 0.0 0.0 0.0 0.83 0.0 0.5—(say). Here, *check*(*S*) becomes *False*, as all the slots pertaining to the option being served are not filled up. So, *TR*_*c*_ = −5 * (2 − 0.83), i.e., a high penalty of −5.85.

- **Intent meta-policy**: *TR*_*i*_ for the intent meta-policy at every time-step (*TR*(*s*, *i*, *s*′)) of the conversation is:
  $$\begin{array}{c} TR_{i}=\{\begin{array}{c} (w_{1}*\|\vec{S_{i}^{\prime }}{\|}_{1}-\|\vec{S_{i}}{\|}_{1},\text{if}\;i=\text{correct}\;\text{option}\\ -w_{1},\;\;\;\;\text{if}\;i=\text{incorrect}\;\text{option} \end{array} \end{array}$$
  where $\|\vec{S_{i}^{\prime }}{\|}_{1}$ represents state vector *S*′ after completing subtask *i*. $\|\vec{S_{i}}{\|}_{1}$ represents state vector *S* while beginning to serve intent *i*.

### Case study

Let $\vec{S_{i}}$ be—0 1 0 0 0.57 0.0 0.0 0.0 0.0 0.5—(say). Let’s say VA (top-level hierarchy) picks up option *i* = 2. After, the low-level hierarchy completes processing this option/subtask *i*, the state $\vec{S_{i}^{\prime }}$ becomes—0 1 0 0 0.74 0.87 0.93 0.0 0.83 0.79—(say). Here, *TR*_*i*_ = 5 * (3.37 − 0.57), i.e., a high positive reward of 14. Now, let’s say VA (top-level hierarchy) picks up an incorrect option *i* = 1 (say) at state $\vec{S_{i}}$—0 1 0 0 0.57 0.0 0.0 0.0 0.0 0.5—(say). Then *TR*_*i*_ = −5, i.e., a high penalty of −5.

- **Sentiment based reward**: The sentiment based reward (SR) which includes *repetition*, *interruption* and *user satisfaction* at every time step of the controller and intent meta policies based on the sentiment tag identified are as follows:
  $$\begin{array}{c} SR\left(s,a/i,s^{\prime }\right)=\{\begin{array}{cc} w_{1}*S_{z}, & \text{if}\;tag=+ve\\ -w_{1}*\left(1-S_{z}\right), & \text{if}\;tag=-ve\\ S_{z}, & \text{otherwise}\; \end{array} \end{array}$$
  The proposed sentiment based immediate rewards banks on the fact that it utilizes user information in the form of sentiment scores that doesn’t involve any manual labeling of the reward or the reward function once a sentiment classifier/detector is ready. Also, it doesn’t require any prior domain knowledge and can be easily generalized to other domains.

### Case study

Here the *S*_*z*_ variable corresponds to the last variable of the state space. So, let’s say state $\vec{S}$ (either for meta or controller policies) be—0 1 0 0 0.74 0.87 0.93 0.0 0.83 0.79—and the sentiment identified be positive. Then *SR* = 5 * 0.79, i.e., a high positive reward of 3.95. Let state $\vec{S}$ be—0 1 0 0 0.74 0.87 0.93 0.0 0.83 0.36—(say) and the sentiment identified be negative. Then *SR* = −5 * (1 − 0.36), i.e., a penalty of −3.2. Now, let state $\vec{S}$ be—0 1 0 0 0.74 0.87 0.0 0.0 0.83 0.5—(say) and the sentiment identified be neutral. Then *SR* = 0.5, i.e., a small positive reward of 0.5.

Thus, the proposed reward at every time-step of the conversation at different hierarchies is:

$$\begin{array}{c} R=TR+SR \end{array}$$

### Case study

Fig 2 shows the architectural diagram of the proposed Hierarchical DRL agent fused with sentiment. The working of this end-to-end system is described as follows: For eg., let the conversation start with the user asking the VA *Which restaurant can I book for two people for today?*. This query of the user is passed through several components of the Natural Language Understanding module comprising of Intent Classification (IC), Slot-Filling (SC) and Sentiment Classification (SC) to extract relevant information and semantics from the user input to be processed by the VA. The IC module (described below) takes as input this user query and returns the corresponding intent of this utterance which is restaurant_info (for this particular utterance). Similarly, this user utterance is also processed through the SF module (described below) which extracts relevant and useful information in the form of slots which are no. of people = two, date = today. Along with this, it is also passed through the SC module to identify the user behavior in terms of sentiment associated with its query which is neutral (for this particular utterance). Now, these extracted information i.e., user semantics and behavior are updated in the state space of the VA (described above). Based on the current or updated state space, the top-level hierarchy of the VA picks up the relevant option (not known to the user) to process the identified intent or sub-task by the low-level hierarchy (say *i*_2_ here refer to the option of serving intent restaurant_info). The low-level hierarchy now picks up a primitive action in order to communicate with the user to serve the option/sub-task in control (option picked up by the top-level hierarchy). Let’s say the VA picked up the action of ask(category), in order to elicit the information from the user. This picked up action is passed through the Natural Language Generation (NLG) module (described below). The NLG module converts the VA’s action to natural language text for it to be presented to the user in the form of System or VA’s response (let’s say Which category restaurant are you looking for? be the response of the VA). This concludes one time-step of the user-VA interaction. Similarly, the conversation continues until the sub-task(s) is completed and the conversation terminates.

## 5 Experimentation details

Natural Language Understanding (NLU) module comprising of Intent Classification (IC), Slot-Filling (SF) and Sentiment Classification (SC) has been pre-trained on the modified bAbI dataset. We trained separate deep learning models for IC, SF and SC on the developed SentiVA dataset to curate the NLU module for a *to and fro* communication between the VA and the user.

### Training and testing

Training RL algorithms requires feedbacks in the form of consequences from the environment which is users in our case. However, interacting with real users for training is highly expensive and sometimes infeasible (for large number of training episodes). Therefore, we have developed a pseudo-environment i.e., a user simulator that is based on a pseudo-random generator to mimic the confidence values and output from the SF, SC and IC modules, respectively, for different intents in control. This is used as an input to the state space of different policies at different levels of hierarchies. This environment and training procedure are curated to represent a real SF, SC and IC as closely as possible and expedite the process of training as much faster and robust to random noises that might exist in a NLU module. This gives the trained DM module the flexibility to be reused and generalize to any other state since it has not been trained on a particular corpus or conversational data for a task, thereby prohibiting it to learn features and policies specific to a corpus. At the beginning of each episode/dialogue the simulator is initiated with a goal consisting of multiple intents out of the four intents with each of them having pre-defined entities and its values mentioned above. The goal remains unchanged till the completion of the initiated multi-intents. However, new goals can be added after no sub-task remains to be completed by the VA depending on the user requirement. To incorporate user sentiment in the simulation phase, we maintain a record or a statistic for every VA action that shows how many times a particular entity has been queried by the VA during the course of a particular dialogue. This is done to counter *repetition* from the VA’s side, as users exhibit strong sentiment when repeatedly asked about a certain entity. Also, after relevant slots have been filled by the VA for a particular sub-task, we maintain a threshold of maximum 3 time-steps for the VA to provide suitable options to the user for it to be satisfied and exhibit positive sentiment. After which, the user sentiment automatically switches to positive in order for the dialogue/episode to terminate. Based on these factors, confidence values are generated using the pseudo-environment to emulate user behavior as an input to the state space at different hierarchies. Later, the learned policy which is trained on the pseudo-environment is tested against real IC, SC and SF modules trained on the dataset discussed above. Thus, real SC, IC and SF modules are integrated with the system replacing the randomness from state space of all the policies, thereby incorporating natural language to test the robustness of the policy learnt. The rest of the system functions exactly in the same manner as described during training enabling slot-constraint, user sentiment and optimally completing the subtasks for a successful dialogue conversation. Algorithm 1 shows the procedure to train the Hierarchical Dialogue Manager with Task Success and Sentiment based Immediate Rewards.

**Algorithm 1** Proposed Hierarchical Learning Algorithm with Task Success and Sentiment based Immediate Rewards (SR+TR)

1: **Initialize**: Set of Deep-Q-Networks (Intent and Controller) with replay memories *M*^(*i*,*ss*)^ and *M*^(*c*,*i*,*ss*)^, action-value functions *Q*^(*i*,*ss*)^ and *Q*^(*c*,*i*,*ss*)^ with random weights *θ*^(*i*,*ss*)^ and *θ*^(*c*,*i*,*ss*)^, and target action value functions ${\hat{Q}}^{\left(i,ss\right)}$ and ${\hat{Q}}^{\left(c,i,ss\right)}$ with weights ${\hat{\theta }}^{\left(i,ss\right)}=\theta^{\left(i,ss\right)}$, ${\hat{\theta }}^{\left(c,i,ss\right)}=\theta^{\left(c,i,ss\right)}$

2: **Initialize**: Sumtrees with maximal priority for replay memories

3: **repeat**

4:  Reset environment, initialize *S*^(*i*,*ss*)^ and *S*^(*c*,*i*,*ss*)^ states where *ss* = sentiment score

5:  *r*^*e*^ = 0, $r_{i}^{ss}=0$

6:  **repeat**

7:   intent_option (i) = *argmax*_*i*∈*I*_ *Q*^(*i*,*ss*)^(*S*^(*i*,*ss*)^;*θ*^(*i*,*ss*)^)

8:   $r^{i_{q}}=0$, $r_{c}^{ss}=0$

9:   **repeat**

10:    *a* = *argmax*_*a*_ *Q*^(*c*,*i*,*ss*)^(*S*^(*c*,*i*,*ss*)^;*θ*^(*c*,*i*,*ss*)^)      ⊳ e.g., *ϵ* greedy

11:    Execute action *a* and observe task reward, $r^{i_{q}}$, sentiment reward, $r_{c}^{ss}$, and next state, *S*^′(*c*,*i*,*ss*)^

12:    Append transition $\left(S^{\left(c,i,ss\right)},a,r^{i_{q}}+r_{c}^{ss},S^{\prime (c,i,ss)}\right)$ to *M*^(*c*,*i*,*ss*)^

13:    *E*^(*c*,*i*,*ss*)^ ← sample random mini-batch of experiments from *M*^(*c*,*i*,*ss*)^ based on maximum priority *P*_*j*_

14:

$$y_{c,i,ss}=\{\begin{array}{cc} r^{i_{q}}+r_{c}^{ss}, & \text{if}\;a\;\text{is}\;\text{terminal}\\ r^{i_{q}}+r_{c}^{ss}+\gamma max_{a\in A^{\left(c,i,ss\right)}}{\hat{Q}}^{\left(c,i,ss\right)}\left(S^{\prime (c,i,ss)},a^{\prime };{\hat{\theta }}^{\left(c,i,ss\right)}\right), & \text{otherwise} \end{array}$$

15:    Update transition priority *P*_*j*_ = |*y*_*c*,*i*,*ss*_|

16:    Gradient descent step on (*y*_*c*,*i*,*ss*_ − *Q*^(*c*,*i*,*ss*)^(*S*^′(*c*,*i*,*ss*)^, *a*′;*θ*^(*c*,*i*,*ss*)^))^2^ using *E*^(*c*,*i*,*ss*)^

17:    Reset ${\hat{Q}}^{\left(c,i,ss\right)}=Q^{\left(c,i,ss\right)}$ every *C* steps

18:    $r^{e}+=r^{i_{q}}+r_{c}^{ss}$, *S*^(*c*,*i*,*ss*)^ = *S*^′(*c*,*i*,*ss*)^

19:   **until** *a* is the terminating action for intent_option *i*

20:   Append transition $\left(S^{\left(i,ss\right)},i,r^{e}+r_{i}^{ss},S^{\prime (i,ss)}\right)$ to *M*^(*i*,*ss*)^

21:   *E*^(*i*,*ss*)^← sample random mini-batch of experiments from *M*^(*i*,*ss*)^ based on maximum priority

22:

$$y_{i,ss}=\{\begin{array}{cc} r^{e}+r_{i}^{ss}, & \text{if}\;i\;\text{is}\;\text{terminal}\\ r^{e}+r_{i}^{ss}+\gamma max_{i\in I^{\left(i,ss\right)}}{\hat{Q}}^{\left(i,ss\right)}\left(S^{\prime (i,ss)},i^{\prime };\theta^{\left(i,ss\right)}\right), & \text{otherwise} \end{array}$$

23:   Update transition priority *P*_*j*_ = |*y*_*i*,*ss*_|

24:   Gradient descent step on (*y*_*i*,*ss*_ − *Q*^(*i*,*ss*)^(*S*^′(*i*,*ss*)^, *i*′;*θ*^(*i*,*ss*)^))^2^ using *E*^(*i*,*ss*)^

25:   Reset ${\hat{Q}}^{\left(i,ss\right)}=Q^{\left(i,ss\right)}$ every *C* steps

26:   *S*^(*i*,*ss*)^ = *S*^′(*i*,*ss*)^

27:  **until** no query left and no new query comes in

28: **until** convergence      ⊳Given number of episodes completed

### Case study for Algorithm 1

Let’s say, we begin with *S*^*i*,*ss*^ = [1 0 0 0 0.7 0.0 0.0 0.0 0.0 0.5] and *S*^*c*,*i*,*ss*^ = [0 0 0 0 0.7 0.0 0.0 0.0 0.0 0.5], *r*^*e*^ = 0, $r_{i}^{ss}=0$ in steps 4 and 5. In step 7, the top-level hierarchy of the VA picks up the most probable option *i* (say *i* = 2) conditioned on the state *S*^*i*,*ss*^ and parameters *θ*^*i*,*ss*^ of the *Q*^(*i*,*ss*)^ action-value function. Now, this picked up option is presented to the state space of the controller policy which now becomes *S*^*c*,*i*,*ss*^ = [0 1 0 0 0.7 0.0 0.0 0.0 0.0 0.5]. The TR and SR for controller policy are set to zero. In step 10, the low-level controller policy picks up the most likely primitive action, *a* (say *a* = 3), conditioned on the state *S*^*c*,*i*,*ss*^ and parameters *θ*^*c*,*i*,*ss*^ of the *Q*^(*c*,*i*,*ss*)^ action-value function. This action *a* = 2 is executed in the environment by the VA. The consequences observed are the next state, *S*^′(*c*,*i*,*ss*)^ = [0 1 0 0 0.7 0.0 0.83 0.0 0.0 0.5] (say), TR, $r^{i_{q}}=3.15$ and SR, $r_{c}^{ss}=0.5$. So, the final reward, *r*_*c*_ for this transition becomes $r_{c}=r^{i_{q}}+r_{c}^{ss}=3.15+0.5=3.65$. This transition is appended in the replay memory *M*^*c*,*i*,*ss*^ in step 12. A mini-batch (say batch size = 32) of such experiences are sampled out from the memory based on maximum priority *P*_*j*_ in step 13. In step 14, true action-value estimates of these samples are calculated in order to train the Deep Q-networks. So, if for a sample (say the above sample), action *a* is the terminating action, then its true action-value estimate becomes, $y_{c,i,ss}=r^{i_{q}}+r_{c}^{ss}$. Here, clearly *a* = 3 is not the terminating action. Otherwise, the current estimate of the action is obtained from ${\hat{Q}}^{\left(c,i,ss\right)}=2.66$ (say) action-value function. This is scaled with the discount factor *γ* and added to its TR and SR. So, the true estimate of the above sample now becomes *y*_*c*,*i*,*ss*_ = 3.15+ 0.5+ 0.7 * 2.66 = 5.51. Now, the error of all the samples of the mini-batch is calculated, i.e., the difference between the true estimate and the current estimate from *Q*^(*c*,*i*,*ss*)^. Let’s say for the above sample, the current estimate from *Q*^(*c*,*i*,*ss*)^ is 3.02. So, the error is of amount 2.49 for this particular sample. The cumulative squared error of all the samples of the mini-batch is back-propagated in the Deep Q-Network *Q*^(*c*,*i*,*ss*)^ using the gradient descent algorithm in order to update the weights of all the parameters accordingly to learn a desired behavior in step 16. After every C steps (say 100), the weights of ${\hat{Q}}^{\left(c,i,ss\right)}$ are equalized with the current estimate *Q*^(*c*,*i*,*ss*)^ in step 17. In step 18, *r*^*e*^ is updated as $r^{e}=r^{e}+r^{i_{q}}+r_{c}^{ss}=0+3.15+0.5=3.65$. The next state *S*^′(*c*,*i*,*ss*)^ becomes the current state *S*^(*c*,*i*,*ss*)^. This process of the controller policy continues until it picks up the terminating action for a given subtask *i* (i = 2 here). After the given subtask is completed by the controller policy, the current state *S*^(*c*,*i*,*ss*)^ becomes [0 1 0 0 0.7 0.96 0.83 0.0 0.88 0.8] (say) and *r*^*e*^ becomes 15.63 (say). Based on this, the next state *S*^′(*i*, *ss*)^ of top-level policy becomes [0 0 0 0 0.7 0.96 0.83 0.0 0.88 0.8], SR, $r_{i}^{ss}=0.8$. So, the final reward, *r*_*f*_ for this higher-level transition becomes $r_{f}=r^{e}+r_{i}^{ss}=15.63+0.8=16.43$. This transition is appended in the replay memory *M*^*i*,*ss*^ in step 20. A mini-batch (say batch size = 32) of such experiences are sampled out from the memory based on maximum priority, *P*_*j*_, in step 21 for the top-level policy. In step 22, true action-value estimates of these samples are calculated in order to train the Deep Q-networks. So, if for a sample (say the above sample), option *i* is the terminating option, then its true action-value estimate becomes, $y_{i,ss}=r^{e}+r_{i}^{ss}$. Here, clearly *i* = 2 is not the terminating option. Otherwise, the current estimate of the option is obtained from ${\hat{Q}}^{\left(i,ss\right)}=10.19$ (say) action-value function. This is scaled with the discount factor, *γ*, and added to its TR and SR. So, the true estimate of the above sample has now become *y*_*i*,*ss*_ = 15.63 + 0.8 + 0.7 * 10.19 = 23.56. Now, the error of all the samples of the mini-batch is calculated, i.e., the difference between the true estimate and the current estimate from *Q*^(*i*,*ss*)^. Let’s say for the above sample, the current estimate from *Q*^(*i*,*ss*)^ is 18.52. So, the error is of amount 5.04 for this particular sample. The cumulative squared error of all the samples of the mini-batch is back-propagated in the Deep Q-Network, *Q*^(*i*,*ss*)^, using the gradient descent algorithm in order to update the weights of all the parameters accordingly to learn a desired behavior in step 24. After every C steps (say 100), the weights of ${\hat{Q}}^{\left(i,ss\right)}$ are equalized with the current estimate, *Q*^(*i*,*ss*)^, in step 25. In step 26, the next state *S*^′(*i*, *ss*)^ becomes the current state *S*^(*i*,*ss*)^. This process continues until no query or subtask is left by the VA to be processed and no new query comes in. Finally, the outer loop in step 28 terminates after the given number of episodes completes execution.

### Intent Classification (IC) module

**Fig 3:**
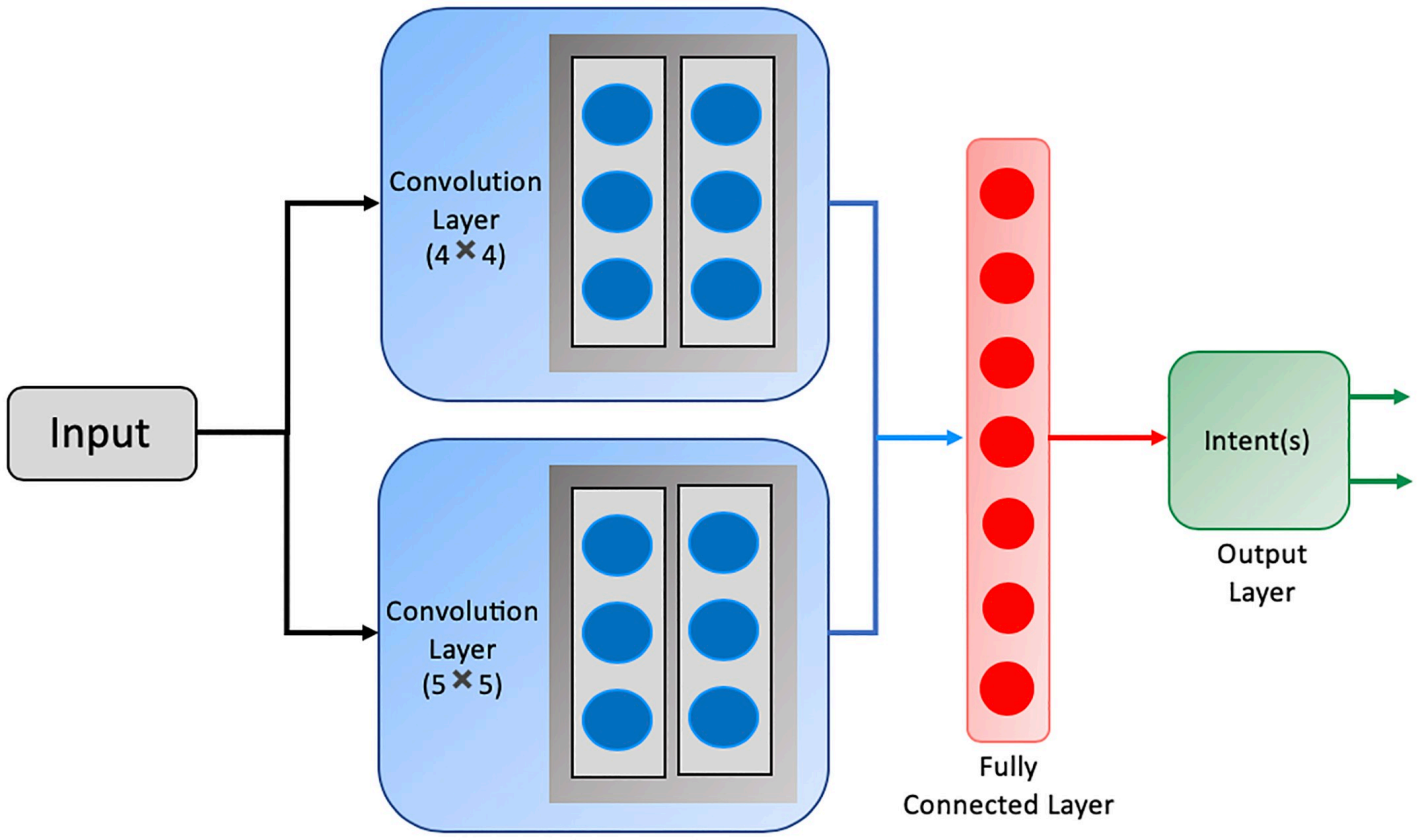
The architectural diagram of Intent Classifier (IC) module.

The task of this module is to identify or predict one or more of the intents from the user’s utterance. Thus, its objective is to maximize the conditional probability of intent(s) *i* given *x*.

$$\begin{array}{c} P\left(i|x\right)=\prod_{q=1\cdots n}P\left(i_{q}|x\right) \end{array}$$

where *n* represents the number of intents in a domain. For this, a two layer Convolutional Neural Network (CNN) based deep learning model has been trained. The input to the network is the word embeddings of the corresponding words in the utterance. GloVe word embedding [39] of dimension 300 has been used to represent words (used for SF and SC modules as well). CNNs of kernel size 4 and 5 with 64 feature maps are used with softmax activation at the final layer for classification. Thus, this module identifies one or more of the intents at a time which is the input to the state space of the intent meta-policy. Fig 3 shows the architectural diagram of the IC module.

### Case study

The IC module takes as input the user utterance at every time-step. It outputs one of the intents from the set of intent labels present in the dataset corresponding to the utterance. For eg., consider an utterance *may i have a table in paris for six in a cheap price range* (say). It outputs the intent as restaurant, i.e., the user wants to know about a restaurant in Paris with a cheap price range that can accommodate reservation for six people. Similarly, for an utterance, *may i have the address and the phone number of resto_bombay_cheap_italian_3star* (say), yields a multi-output of phone number, address.

CNNs are known to be a popular choice for classification task. Here, IC is treated as a classification task. CNN layer learns abstract representation of the phrases reflecting its semantic meaning which finally spans over to the entire sentence. It basically captures abstract n-gram features. Here, by using two extracted layers of filter size 4 and 5, it intends to identify abstract 4-gram and 5-gram features spanning over the sentence to capture context across a longer sentence. The features from both the convolution layers of varying filter sizes learn different kinds of semantic features, which are then concatenated to pass through a fully-connected layer to learn a sentence representation. This representation is then passed through a softmax layer to obtain the classified output or the intent. The motivation is that with a single layer CNN, we might miss semantic information ranging across longer sentences. With more complex model than a two layered CNN, the complexity of the model increases without any significant increase in the accuracy or precision of the classified output. This is also evident through empirical results as shown in Table 3. As seen in the table, with a single layer CNN, the model attained an accuracy of 83.64% whereas with a two layer CNN attained an accuracy of 85.62%, i.e., an increase of about 2%. On the other hand, with a three layer CNN, the model attained an accuracy of 85.86%, i.e., an increase of less than 0.5% compared to its two layer counterpart. Additionally, we also provide results of other models such as Bi-LSTM, GRU etc.

**Table 3:** Quantitative analysis of intent classification module.

Model	Metric	
	Accuracy	F1 Score
GRU	79.85	0.7536
LSTM	80.27	0.7595
Bi-LSTM	82.39	0.7809
CNN (1 layer)	83.64	0.7990
CNN (2 layer)	**85.62**	**0.8096**
CNN (3 layer)	85.86	0.8105

### Slot-Filling (SF) module

To extract relevant information from the user’s utterance in the form of slots, an SF module has been trained. It is a deep learning model which uses a single Bi-directional Long Short Term Memory (Bi-LSTM) Network [40] at its core.

$$\begin{array}{c} \vec{y}=Bi-LSTM\left(\vec{x}\right) \end{array}$$

where $\vec{x}$ is the input word sequence and $\vec{y}$ contains its corresponding slot labels. The number of hidden units used for the Bi-LSTM is 90 with the softmax activation at the final layer. The necessary slots identified, along with the probability scores of the predicted labels are used by state space of both the intent meta and controller policies for further processing.

### Case study

**Fig 4:**
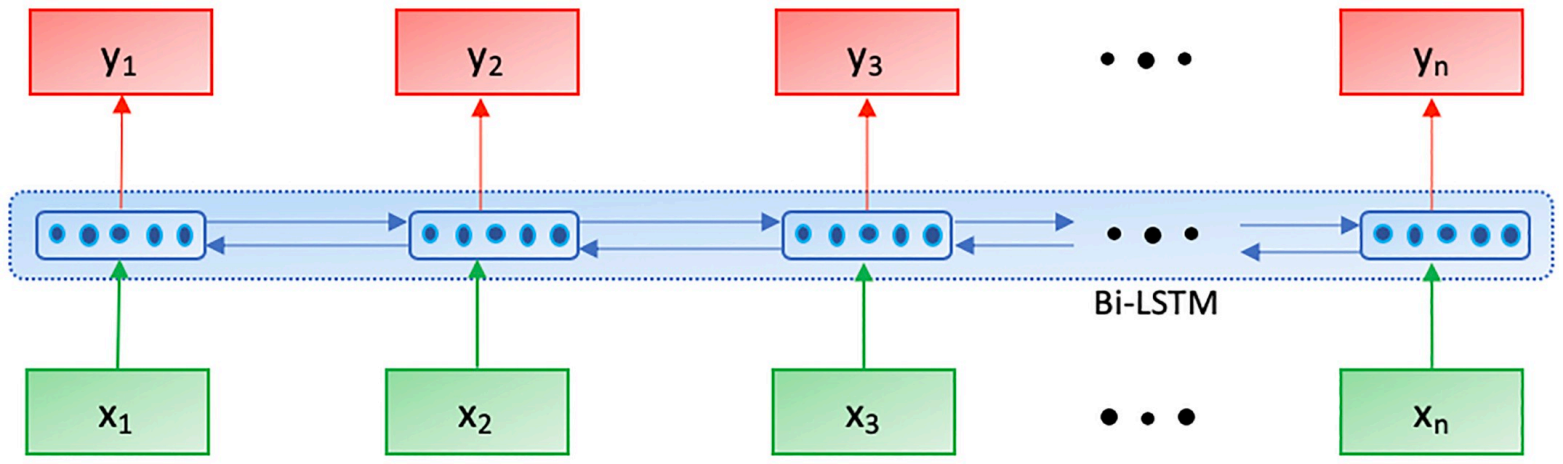
The architectural diagram of Slot-Filling (SF) module.

The SF modules takes as input the entire word sequence in the form of word embeddings and outputs slot labels for each of the words present in the sequence from the set of slot labels present in the dataset. For eg., consider an utterance *may i have a table in a cheap price range in madrid with french food* (say). It gives an output for each of the words in the sequence as “O”, “O”, “O”, “O”, “O”, “O”, “O”, price, “O”, “O”, “O”, location, “O”, cuisine, “O”. Here, “O” refers to as null i.e., no relevant information is present in these respective words. Whereas, labels such as price, location and cuisine provide useful information to the VA related to the user’s preference. So, as seen in Fig 4, *x*_1_, *x*_2_…, *x*_*n*_ refer to each of the words in the user utterance and *y*_1_, *y*_2_…, *y*_*n*_ refer to the corresponding slot labels for each of these words.

### Sentiment Classification (SC) module

To identify the implicit sentiment of an user utterance, an SC module has been trained. Here also, we use a single Bi-directional Long Short Term Memory (Bi-LSTM) Network.

$$\begin{array}{c} \vec{y}=Bi-LSTM\left(\vec{x}\right) \end{array}$$

where $\vec{x}$ is the input sentence representation and $\vec{y}$ contains its corresponding sentiment label. Number of hidden units used for the Bi-LSTM is 90 with the softmax activation at the final layer. The sentiment identified, along with the probability scores of the predicted sentiment labels are used by state space of both the intent meta and controller policies for further processing. Table 4 shows the quantitative analysis of SC module in terms of varying architectures.

**Table 4:** Quantitative analysis of sentiment classification module.

Model	Metric	
	Accuracy	F1 Score
RNN	95.62	0.9517
GRU	96.35	0.9606
LSTM	96.74	0.9641
Bi-LSTM	**98.14**	**0.9807**

For the SC and SF modules, Bi-LSTM have been used. Bi-LSTM is a popular choice while processing sequential information. They are known to capture long-term dependency features across a sequence in both directions i.e., one signal access past information in forward direction while the other access future information in reverse direction. While handling the above two tasks i.e., SC and SF, long-term context throughout the sequence is of utmost importance. Whereas, RNNs suffer from vanishing gradient problems and is unable to capture long-term context in practicality. Bi-LSTMs also has the advantage to learn how and when to forget unnecessary information and when not to use gates in their architecture. Whereas GRUs do not make use of any kind of gates in their architecture thereby encompassing all the information throughout a sequence without any filter. This makes the entire learning process complex and heavy-weight.

### Natural language generation

A retrieval based NLG framework has been used that maps the action picked up by the VA to its corresponding natural language to present to the user. Similarly, predefined sentence templates with slot placeholders which are replaced by the user goal for a dialogue have been defined for the user responses to present to the VA [1].

### Model architecture

The architectures of the neural network for the policies are as follows: number of nodes in the input layer is equivalent to the size of state space of each policy, followed by one hidden layer with 75 nodes. Number of nodes in the output layer is equivalent to the action set (options or primitive actions) for each of the policies. The activation function of the hidden layer is ReLU. The Double Deep Q-Network with Prioritized Experience Replay (DDQN-PER) [41] algorithm has been used to train the policies. The other parameters of the DRL model or policies are: discount factor (*γ*) = 0.7, minimum epsilon = 0.15, experience replay size = 100000, batch size = 32. The training is done for 20000 dialogues.

## 6 Results and discussion

The following metrics were used to analyze the performance of various baselines and the proposed framework:

1. *Learning Curve* during training: This gives a visual representation of the learning pattern and growth of the VA during training.
2. *Average Dialogue Length/Turn*: It is basically the average system actions per dialogue. The VA should be able to complete its task in less number of time-steps.
3. *User Satisfaction*: It gives an estimate of the qualitative analysis of the conversations and to determine if the actions picked up by the VA help user attained maximum satisfaction and experience. This is done by analyzing for how many dialogues, the conversation ended on a positive note for the user by monitoring the user sentiment score at the end of the dialogue.

Second and third metrics are computed by taking the average of 100 such executions of the policy during testing with the intents picked up in random. The values reported for the baselines and state of the art model are obtained by taking the mean of the values obtained by executing different intents sequentially (in the same order as the proposed system).

To evaluate the performance of the proposed framework, we compare our model with the following baselines:

- **Flat DRL**: Trained with a single state space encompassing all the intents and slots of a domain collectively without any abstraction or hierarchies;
- **HDRL(TR)**: Contains only task based rewards in the hierarchical framework with different algorithms;
- **HDRL(SR_partial)**: Encompasses only sentiment based rewards in the hierarchical framework to ensure user satisfaction at the end of the conversation without incorporating repetitions and interruptions;
- **HDRL(SR)**: Encompasses only sentiment based rewards in the hierarchical framework to ensure user satisfaction, repetitions and interruptions.

**Fig 5:**
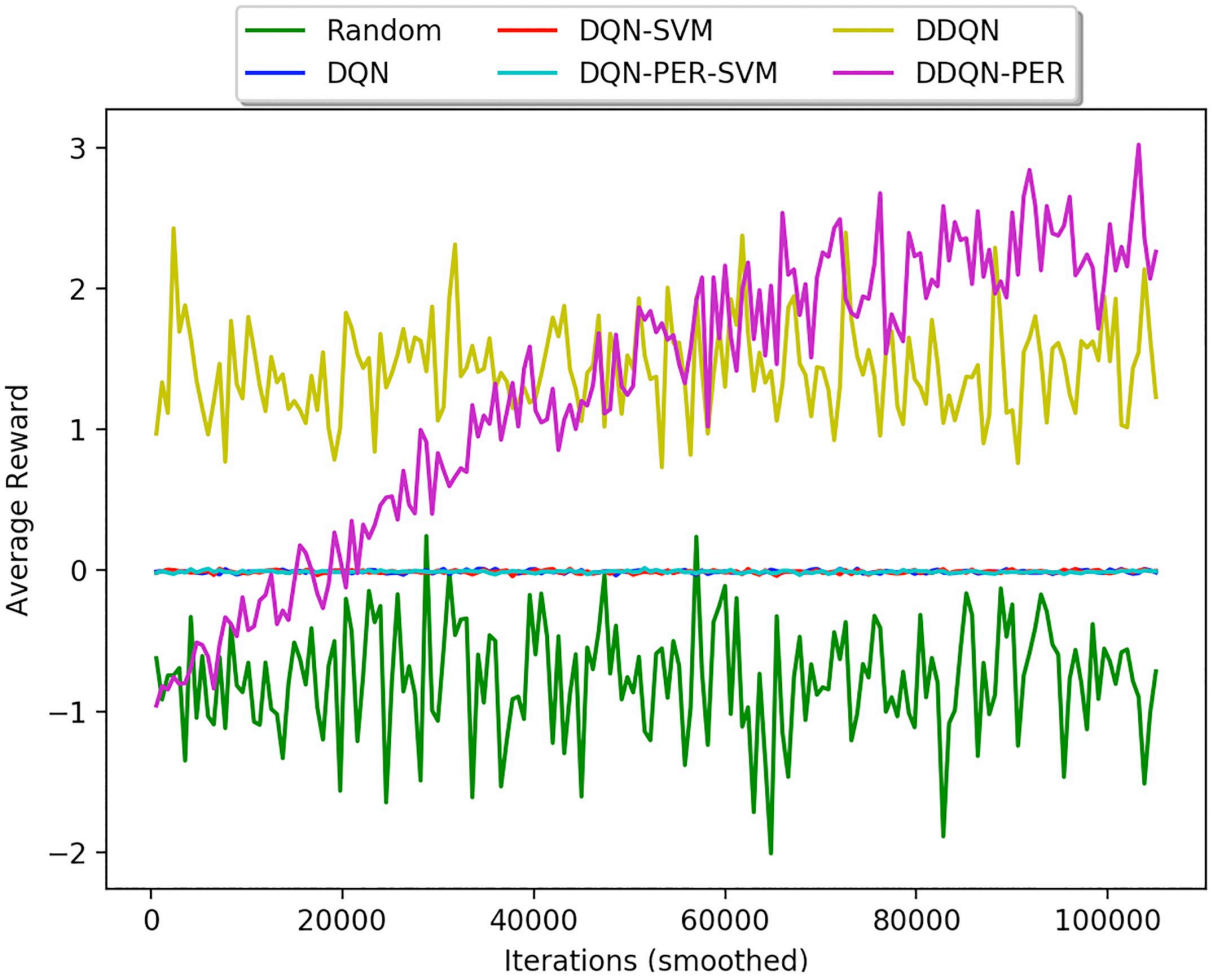
Learning curve of TR based policies during training for different algorithms.

Fig 5 shows the learning curves of different TR based policies with varying algorithms such as Random Agent, DQN [42], DQN-SVM [1], DQN-PER-SVM, DDQN [43], DDQN-PER [41]. As seen from the figure, Random Agent performs the worst compared to all other training algorithms. This is because the random agent takes up random action at every time-step with no learning algorithm guiding the VA. Whereas all the DQN based variations of the algorithms do not converge at all. The policy do not improve over time. This is in lines with [43], where authors demonstrated that DQN has a problem of overestimating the q values, because of the max operation. Whereas DDQN addresses this problem by using a Q-Network (that which is updated) to select the action *a* for which the target network computes the estimated reward. As seen in Fig 5, DDQN performs comparatively better than all its DQN counterparts. However, it is observed that DDQN-PER perfroms the best amongst all the learning algorithms as the concept of PER stresses more on such samples whose error is large compared to other experiences. Thus, DDQN-PER is used as the learning algorithm for all the remaining experiments.

**Fig 6:**
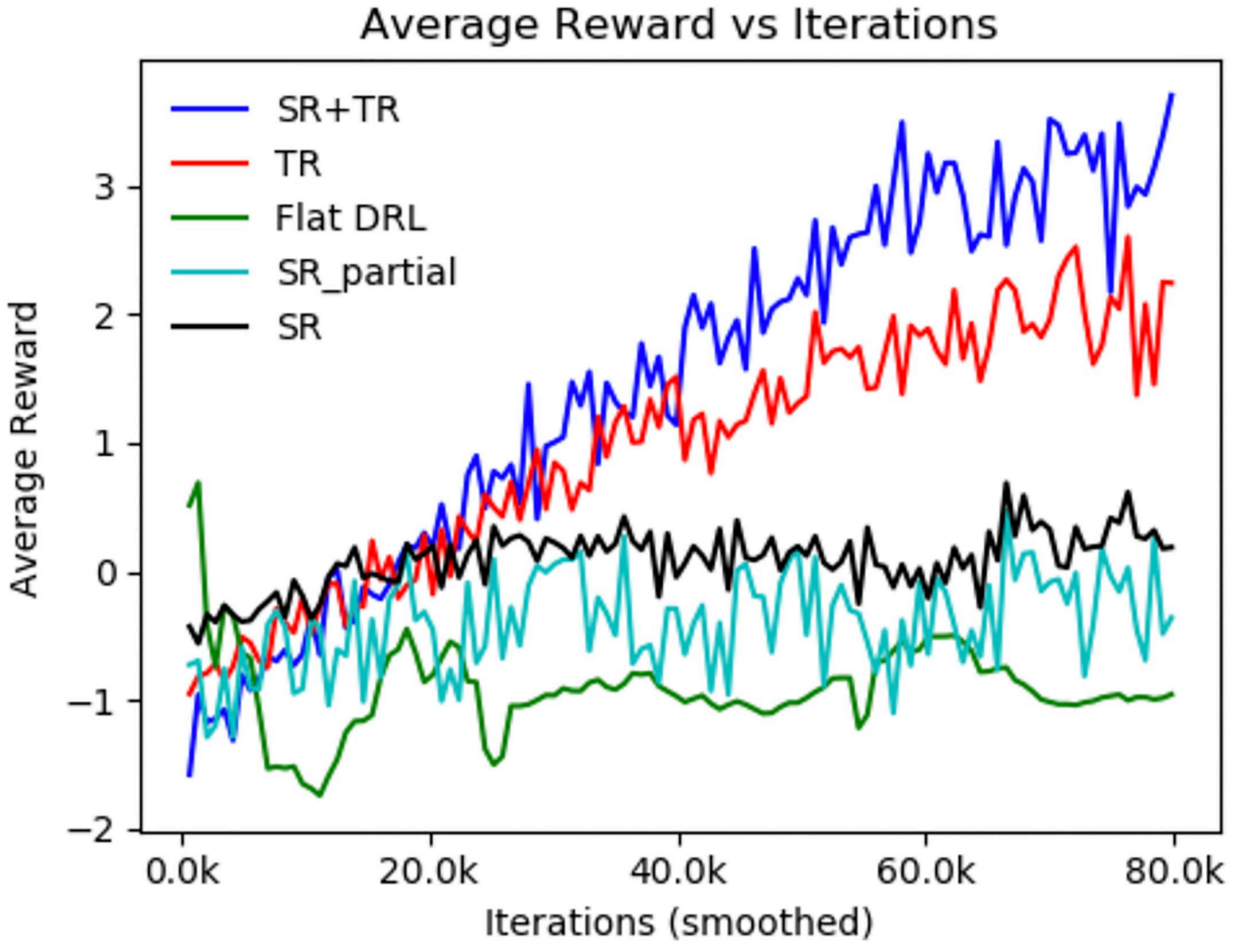
Learning curve of various policies during training.

**Fig 7:**
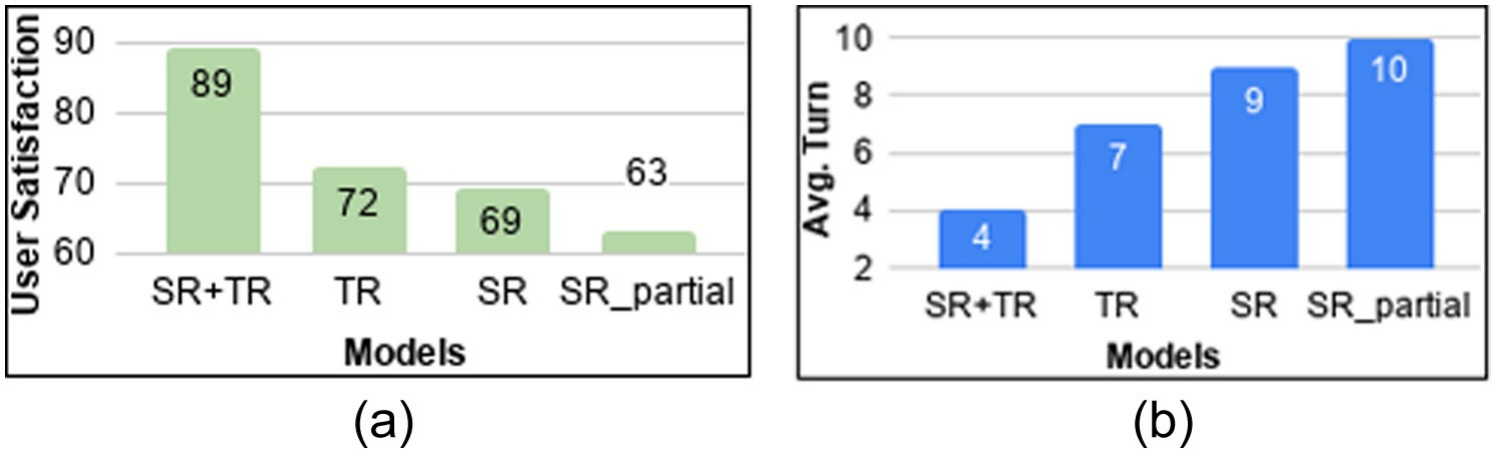
Performance of the VAs during testing with different measures: (a) *User Satisfaction*, (b) *Avg. Turn*.

Fig 6 shows the learning curves of different models during training. It is seen that the flat DRL policy does not improve or learn over time due to the increased complexity in the flat state space encompassing all the intents and slots together without any abstraction or hierarchies. Fig 7a and 7b show the performance of all these policies during testing (with 100 dialogues) in terms of user satisfaction and average turn. Here, user satisfaction includes successful task completion along with positive gratification from the user. All the reported results are statistically significant [44] at 5% significance level. Out of all the policies, HDRL(SR+TR), i.e., the combination of both the rewards yielded the best results and efficient convergence of the policy as visible. This is due to the fact that by taking into account the user sentiment, the VA is able to avoid unnecessary actions to make the conversation more effective. The importance of including repetitions and interruptions along with user satisfaction can be realised by viewing the difference between *SR_partial* and *SR*. This is because by incorporating repetition, the VA encompassed and learned more data points leading to the VA taking lesser time-steps to complete the conversation with higher user satisfaction. Detailed analysis of the policies revealed that with *TR* alone, VA wasn’t able to consider the sentiment of the user thereby taking more number of dialogue turns to complete a given sub-task. Whereas, with *SR* alone, the user wasn’t necessarily giving a negative sentiment to an irrelevant slot query in multi-intent scenario leading to unnecessary VA actions.

### Statistical significance test

For statistical significance test, we have performed Welch’s t-test [44]. The test is performed at 5% significance level. Welch’s t-test is conducted between SR+TR and the remaining models and the results are reported in Table 5. All the p-values reported in Table 5 are less than 0.05. These values establish that improvements obtained by SR+TR models over other baseline are statistically significant.

**Table 5:** p-values reported by Welch’s t-test on comparing our proposed SR+TR model with other models.

Model	Avg. Turn	User Satisfaction
TR	0.029	3.04*e*^−12^
SR	5.36*e*^−4^	4.97*e*^−15^
SR_partial	4.0*e*^−6^	1.89*e*^−16^

### Human evaluation

**Fig 8:**
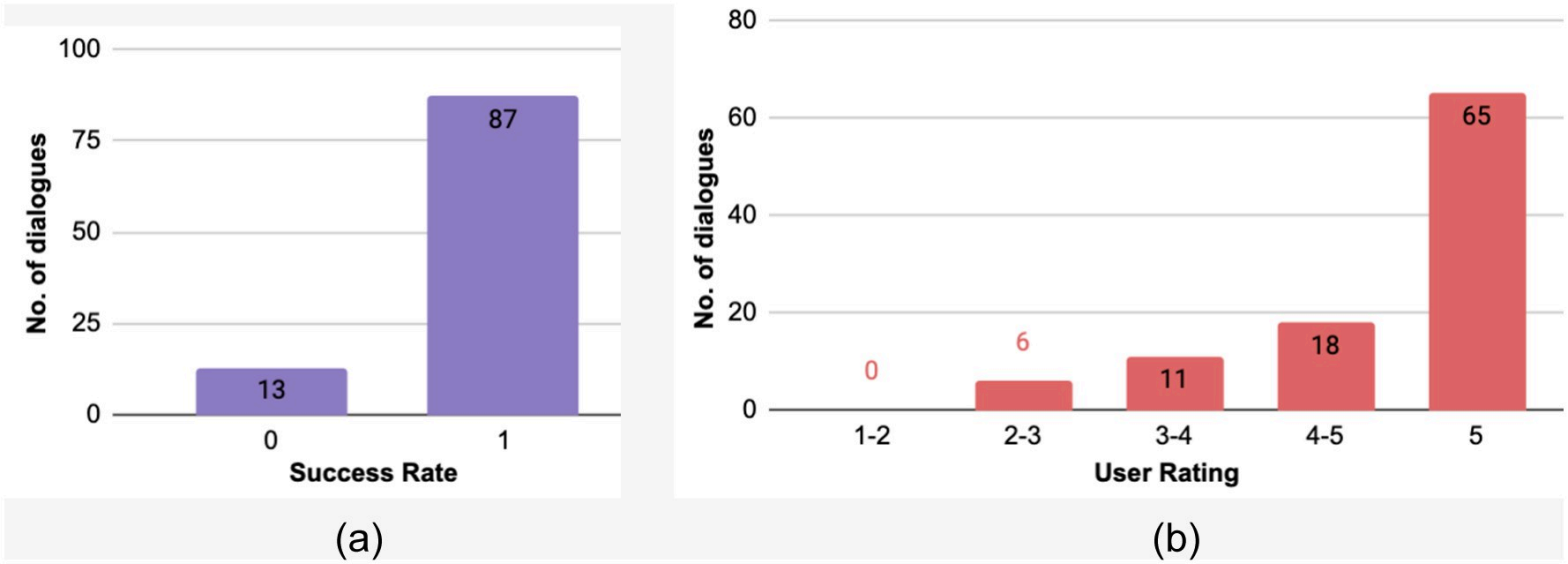
Performance of the VAs tested with human evaluators: (a) success rate based on binary marking schema, (b) Distribution of user-ratings based on variable marking schema for SR+TR.

Three human users from the authors’ affiliation were asked to rate the quality of the dialogues generated from the SR+TR VA. The users were presented with 100 simulated dialogues during testing. For each of the dialogues, users were then asked to rate the general quality of the conversation and the VA on two marking schema: (i) Rating the dialogue on a scale of 1 (worst) to 5 (best) to get a detailed marking score based on *coherence*, *sentiment awareness* and *naturalness*. By *coherence*, we mean that the VA should ask questions or provide information based on the query of the user. The users’ need and the VAs’ actions should be coherent. *Sentiment awareness* refers to whether VA takes into consideration sentiment of the user during the dialogue and whether the conversation ends on a positive note for the user. *Naturalness* refers to the users’ view on how suitable or successful the VA can be in its endeavor without much difficulty in terms of achieving the user goal. (ii) Binary scoring of 1 (good) or 0 (bad) to evaluate whether the conversation was successful or not. Fig 8b shows the subjective evaluation in terms of user rating based on the first marking schema. Fig 8a presents the performance of the VAs against human evaluators in terms of the success rate.

### Error analysis

**Fig 9:**
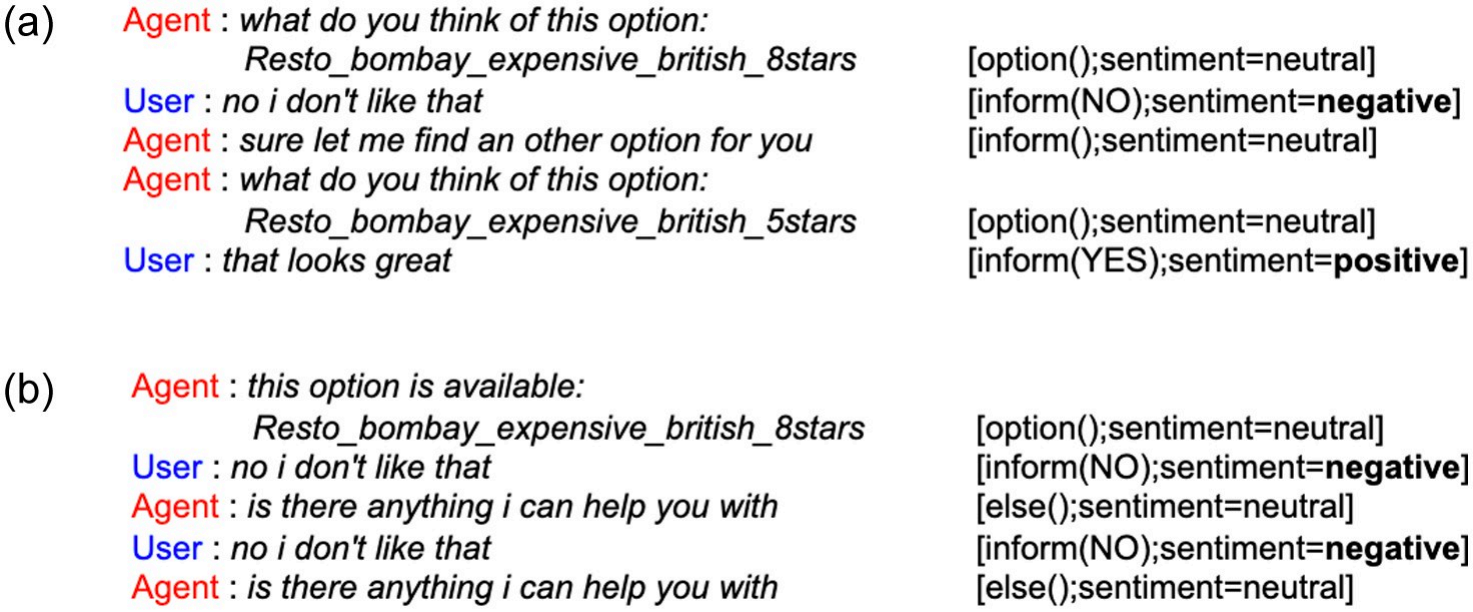
Performance of the VAs during testing: (a) SR+TR, (b) TR.

Some instances from the chat transcript to portray the differences amongst the baseline and proposed policies during testing are shown in Fig 9. As is evident, when the *SR+TR* VA detected a negative sentiment from the user, irrespective of it successfully filling all the irrelevant slots, it had the capability to recover from such a scenario by executing a more efficient strategy. There was no rule-based strategy to force the model to pick up actions after encountering such a situation, but the model learned these fine differences by itself with the help of robust reward functions. Whereas, the *TR* VA falls in a loop, unable to revive itself from such a scenario, thus, stressing the role of incorporating sentiment for every sub-task in the hierarchical value functions.

Also detailed observation and analysis of the proposed HDRL (SR+TR) system revealed various scenarios where the system falters which are discussed as follows:

- **Sentiment Identification Error**: Sentiment score inputs to the state space of the intent meta and controller policies are managed by the SC module in order to achieve maximum user satisfaction. A mis-classification of the intended implicit sentiment due to the limitation of the SC module leads to the Dialogue Manager ignoring or misjudging the user’s sentiment towards attainment of user gratification. For e.g., for an user utterance *do you have something else*, the sentiment was incorrectly identified as neutral instead of negative, that leads to the Dialogue Manager ignoring the sentiment of the user; thus, making the user dissatisfied by the actions picked up by the VA further.
- **Intent Identification Error**: Inputs to the state space of the intent meta-policy is managed by the IC module in terms of multiple subtasks to be completed in order to achieve the user goal. A mis-classification of the intended intent due to the ambiguous user utterance or the limitation of the IC module leads to the Dialogue Manager serving a wrong intent. For e.g., for an user utterance *I need a table for two*, the intent was incorrectly identified as restaurant_info instead of restaurant_book, that leads to the Dialogue Manager executing a wrong controller policy based on the option picked up by the intent meta-policy; thus, making the user dissatisfied by the information provided by the VA.
- **Slot-filling Error**: Similarly, a mis-identification of the relevant user information in the form of slots leads to the VA taking extra turns to retrieve the correct information thereby increasing dialogue length. For e.g., for an user utterance *I want to eat sea food*, the *cuisine* slot was wrongly identified as sea with a very low confidence. This prompted the VA to confirm the acquired slot from the user as per its controller policy, to which the user denied, thereby taking extra turns to elicit correct information from the user.

Quantitative Analysis of all the above modules with respect to varying architectures are shown in Table 3, 6 and 4 in terms of accuracy and F1-score. As seen from Table 4, the error rate of SC is about 2%, i.e., correct classification of sentiment indeed helps the VA in serving the user with less number of dialogue turns. The error rate of IC on the other hand is about 15% (refer to Table 3), i.e., by using the utterances from the dataset, intents were wrongly classified for significant number of times leading to unsuccessful dialogue conversation and reduced user satisfaction. The SF module has an error rate of almost 19% (refer to Table 6) which is significantly larger. But the VA still has the capability to recover from the errors of SF module by reasking about a particular entity based on its confidence score. This though increases number of dialogue turns but ensures user satisfaction at the end of the conversation.

**Table 6:** Quantitative analysis of slot-filling module.

Model	Metric	
	Accuracy	F1 Score
RNN	74.93	0.7305
GRU	78.66	0.7649
LSTM	79.81	0.7701
Bi-LSTM	**81.52**	**0.7969**

## 7 Conclusion

### Discussion, implication and conclusion

This paper presents a HRL based DM using *Options* framework for managing multi-intent conversations. Sentiment based immediate rewards are incorporated at every time-step of the hierarchical value functions to induce user adaptiveness behavior in the VA. To enable research with these aspects, a novel dataset, SentiVA, is created that contains multi-intent task-oriented dialogue conversations of Restaurant domain annotated with its intent, slot and sentiment labels. A unique representation of Semi-MDP is presented along with novel task-based and sentiment based reward models. These rewards are induced in the hierarchical value functions (*options* here). This paper shows experimentally that sentiment based rewards are necessary to be incorporated along with task based rewards to ensure successful task completion and acquire maximum user contentment by taking into account several notions of sentiment from the user perspective such as successful subtask completion, repetition and interruption.

### Discussion on the compliance with the literature review

There exist varieties of works in the literature that make use of different HRL techniques to develop VAs [23, 25]. However, all these works only incorporate user queries belonging to just one intent/subtask per domain. Also works such as of [1, 3] utilize separate or individual DRL models for each subtask/intent of a domain, thus, creating networks of DRL models for multi-domain conversations. However, in practicality these assumptions and techniques limit the usage of such heavy-weight models. It is to be noted that all these end-to-end frameworks also do not incorporate user sentiment as the guiding factor to the VA. In [10], authors have used only sentiment based immediate rewards in an end-to-end dialogue system for a single intent. But in multi-intent conversations, sentiment alone is not sufficient to learn desired user behavior. Thus, the current study shows how HRL can be employed to provide a learning framework that caters to the requirement of handling various subtasks at the same time while additionally also taking into account other behavioral cues of the user such as sentiment to serve the user in an efficient manner.

### Conclusion

As discussed above, the paper shows that it is crucial to include other behavioral cues of the user such as sentiment to ensure higher user satisfaction and success of such composite task-oriented VAs. The paper demonstrates a methodology to induce sentiment and make VAs user adaptive in the dialogue learning policy by introduction of novel state space and reward models.

### Academic implication

Several works in recent times are focused on developing task-oriented VAs grounded with various aspects such as sentiment, emotion, empathy in several modules of the Dialogue system and so on in order to serve the user efficiently. The proposed approach leverages from the fact that it can be easily adapted for any other domain because of its task-independent methodology and training procedure, thus, stressing the importance of such light-weight models for the complex yet one of the most important modules of the Dialogue system i.e., DM.

### Limitation

However, because of limited training data for multiple intents, the HDRL agent has been trained using a simulator (pseudo-environment). Training the VA with real-time data will surely make it much more diverse and relevant. Also, in the current form, the VA is unable to process an unknown slot or dynamic slot value given by the user. For eg., if the user communicates a preference over say parking, i.e., a slot rarely found and not known to the VA, it deals with such a situation in a very minimalistic way (say reduced user satisfaction) as the VA is not rigged with a robust error-handling strategy in that context.

### Future studies and recommendations

In future, we would like to extend this idea to managing conversations pertaining to multiple intents belonging to multiple domains with the increased level of hierarchy. Also, many chat bots have been deployed over the years but cannot be used across the globe because of language constraints and the range of these facilities thus becomes limited. Deploying the proposed framework to curate VAs in low-resource language will also be addressed in the future work since this will increase its diversity and make it available for many more people. Also, we will focus on incorporating other channels of identifying sentiment in task-based scenarios thus stressing the role of multi-modality. Users do not only relay their queries through text but also use other communication forms such as images. Integrating these multi-modal dimensions of knowledge elicitation is becoming crucial with time and will be addressed in the future work.
